# When Sarcomas Mimic Lymphomas: A BCOR-Rearranged Sarcoma Case Report

**DOI:** 10.7759/cureus.54944

**Published:** 2024-02-26

**Authors:** Ashwin Jitheesh, Ananth Pai

**Affiliations:** 1 Department of Medical Oncology, Kasturba Medical College, Manipal, Manipal, IND

**Keywords:** bcor-rearranged sarcoma, round cell sarcoma, undifferentiated small round cell sarcoma, non-hodgkin’s lymphoma, cervical lymph nodes

## Abstract

This case report describes the diagnostic and therapeutic challenges faced in managing an elderly diabetic man with BCOR-rearranged sarcoma, a rare, aggressive malignancy. The patient presented with neck swelling, initially suspected to be a high-grade lymphoma but later found to be undifferentiated small round cell sarcoma. Further investigations with PET-CT revealed a mass in the lower abdomen, leading us to reconsider the prior diagnosis of non-Hodgkins' lymphoma. Subsequent biopsies from an abdominal deposit indicated a high-grade round cell sarcoma with differentials including BCOR-CCNB3 fusion/BCOR-ITD sarcoma, CIC-DUX4 fusion sarcoma, and EWSR-non-ETS fusion sarcoma. A second opinion from a dedicated oncopathology lab confirmed the diagnosis of BCOR-rearranged sarcoma. The patient underwent exploratory laparotomy and diversion stoma but developed complications post-surgery. Due to the advanced stage and extensive metastases, the patient opted for supportive care due to poor outcomes with treatment. This case underscores the importance of raising awareness and conducting further research to improve the management of rare malignancies like BCOR-rearranged sarcoma.

## Introduction

The *BCOR* gene is found on the X chromosome and produces a protein called BCL6 corepressor [1]. BCL-6 is A POZ/zinc finger transcriptional repressor that is necessary for the development of germinal centers and may impact apoptosis. BCOR-rearranged sarcomas are characterized by alterations involving BCOR genes such as BCOR-CCNB3, BCOR-MAML3, ZC3H7B-BCOR fusion genes, and BCOR internal tandem duplication (ITD)[2]. BCOR-rearranged sarcomas are classified under undifferentiated small round cell sarcomas of bone and soft tissue along with Ewing sarcoma, round cell sarcoma with EWSR1-non-ETS fusions, CIC-rearranged sarcomas [3].

The BCOR-rearranged tumors frequently exhibited spindle cell areas, which appear distinct from most other undifferentiated small round cell sarcomas and share histologic overlap with poorly differentiated synovial sarcoma. These areas are well-defined in intersecting fascicles or blended with the round cell component [4].

Undifferentiated BCOR-CCNB3 fusion sarcoma tends to affect the skeletons of adolescent males [5]. They are usually found in soft tissue and axial or appendicular skeletons. Atypically, they may be found in the paranasal sinus and or lung. A case of a BCOR-CCNB3 sarcoma arising in the pharynx has also been described, albeit in an 18-year-old patient [6]. Neoadjuvant chemotherapy, surgery, and postoperative adjuvant chemotherapy, representing the same strategy employed for Ewing sarcoma, have been recommended as a standard treatment for BCOR-CCNB3 sarcoma [7].

Below is a case of BCOR-rearranged sarcoma presenting as cervical lymphadenopathy with an abdominal deposit in the rectosigmoid colon.

## Case presentation

An elderly diabetic man presented with a right-sided neck swelling that persisted for three months. A prior biopsy revealed a chronic inflammatory lesion. Despite receiving anti-tubercular therapy, he did not experience any improvement in his condition.

A large matted, non-tender firm mass was observed on local examination in the right cervical region. A video laryngoscopy revealed a protruding mass with extrinsic compression near the right pyriform fossa, and a biopsy was taken.

**Figure 1:**
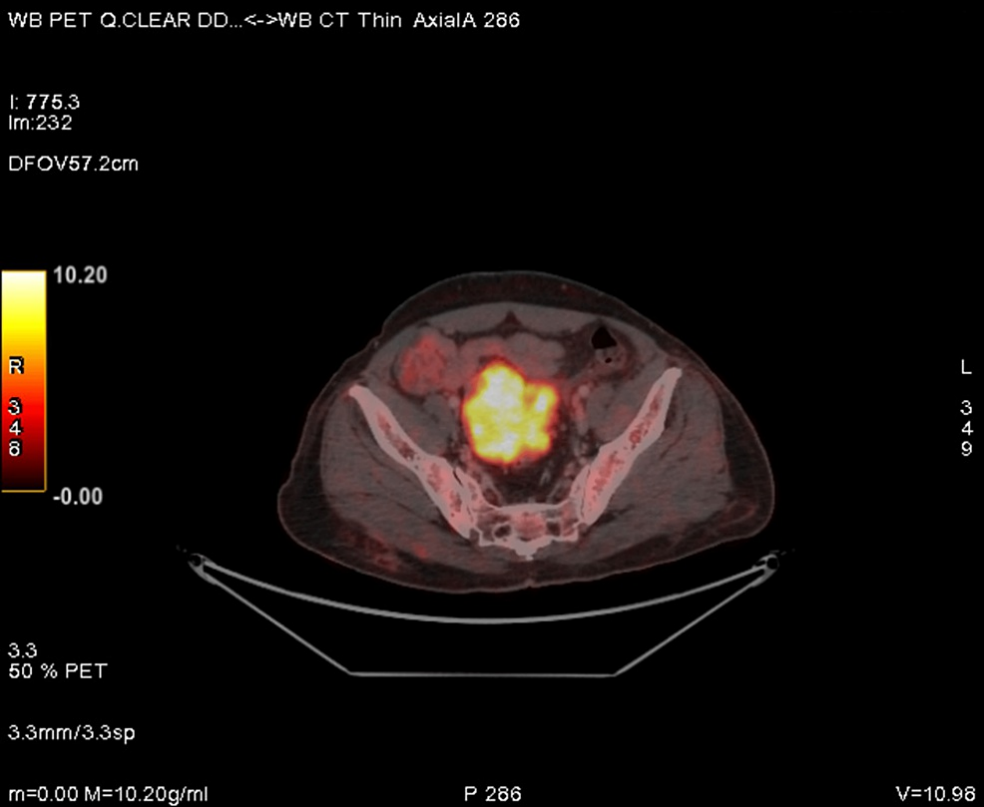
PET-CT showing FDG avid heterogeneously enhancing soft tissue density mass lesion in rectovesical pouch measuring 7.4 × 5.8 × 6.2 cm. FDG: 18-fluorodeoxyglucose; PET/CT: positron emission tomography/computed tomography

**Figure 2:**
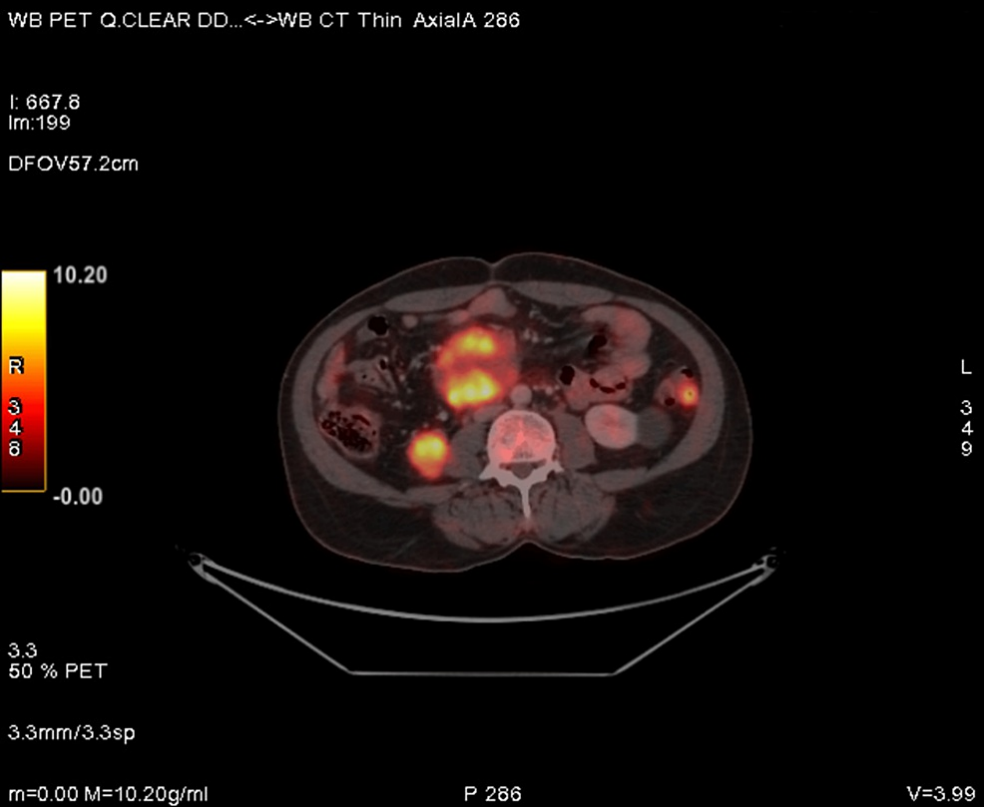
PET-CT showing FDG avid enlarged peri gastric, peri pancreatic, lower para-aortic, mesenteric, and left common iliac lymph nodes. FDG: 18-fluorodeoxyglucose; PET/CT: positron emission tomography/computed tomography

**Figure 3:**
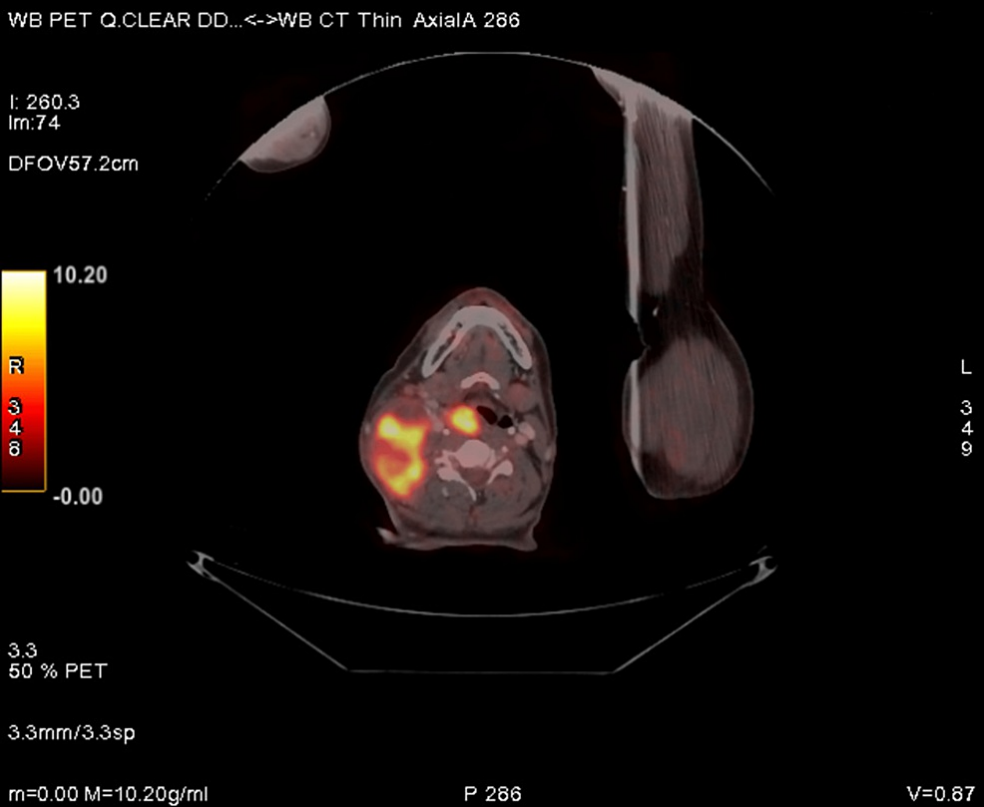
PET-CT showing FDG avid heterogeneously enhancing enlarged right cervical level II, III, IV, Vb, and right retropharyngeal lymph node.

The initial histopathological report suggested a poorly differentiated malignancy suggestive of high-grade lymphoma. He was treated with pre-phase steroids as he had insignificant symptoms pending the immunohistochemistry (IHC) report. A sigmoidoscopy revealed an extrinsic bulge causing luminal narrowing at the rectosigmoid junction, and biopsies were taken for histopathological examination. The IHC confirmed it to be an undifferentiated small round cell sarcoma, and further molecular studies (BCOR, NKX2.2, ET4) were advised. An 18-fluorodeoxyglucose (FDG) positron emission tomography/computed tomography (PET/CT) study showed hypermetabolic enlarged and metastatic lymph nodes above and below the diaphragm (Figures 1-3).

The patient subsequently presented with diffuse abdominal pain radiating to the back and obstructive symptoms suggesting an acute mechanical intestinal obstruction. Before emergent surgery for intestinal obstruction, an IV contrast-enhanced CT abdomen showed a lumen-occluding heterogeneously enhancing mass involving the upper rectum and sigmoid colon with metastatic abdominal lymphadenopathy and peritoneal deposits. The patient underwent an exploratory laparotomy and diversion stoma with loop diversion.

Pathological report update

The dedicated oncopathology laboratory report revealed an undifferentiated round cell sarcoma consistent with a BCOR-rearranged sarcoma. Immunohistochemistry showed tumor cells expressing CD99 (weak), synaptophysin, SATB2, TLE1 (weak), and BCOR. The tumor was immune-negative for NKX2.2, Pax5, CD3, TdT, desmin, chromogranin A, cytokeratin, INSM1, cyclin D1, EMA, CD45, WT1, CD7, CD4 & CD123.

Prognosis and discharge

Given the advanced stage of the disease and the extent of metastases, the patient opted for supportive care.

## Discussion

The case presented here highlights a scenario of BCOR-rearranged sarcoma mimicking lymphoma.

The abovementioned case is an atypical presentation of BCOR-rearranged sarcoma with cervical lymphadenopathy and involvement in the rectosigmoid colon as well as metastatic abdominal deposits. Undifferentiated BCOR-CCNB3 fusion sarcoma tends to affect the skeletons of adolescent males and has a varied anatomic distribution [5]. This case, involving a diabetic man in his late 50s, is quite unusual. Enlarged cervical lymph nodes from lymph node metastasis are also quite rare for sarcoma [8-9].

The initial histopathological report suspected it to be a high-grade lymphoma, then amended to a sarcoma, indicating the diagnostic challenges associated with identifying rare sarcomas. These malignancies can often mimic other tumor types, delaying an accurate diagnosis.

In this case, a second opinion from a dedicated oncopathology lab was crucial in reaching an accurate diagnosis. This emphasizes the significance of utilizing specialized IHC and molecular studies for diagnosing rare malignancies and differentiating sarcoma subtypes. Due to the advanced stage of the disease, extensive metastases, and poor response to treatment, the patient opted for supportive care.

As BCOR-rearranged sarcoma is a rare and relatively newly identified malignancy, research efforts should focus on understanding its pathogenesis, molecular characteristics, and potential targeted treatment options. Clinical trials and collaborative studies can help expand our knowledge and improve outcomes for patients with this sarcoma subtype. Early detection could be essential to patient survival.

## Conclusions

This case report highlights the challenging diagnostic and therapeutic journey of an elderly diabetic man with BCOR-rearranged sarcoma. His unusual presentation with cervical lymphadenopathy, despite the multidisciplinary approach and treatment, led to the disease progressing extensively, leading to palliative care management to enhance the patient's quality of life. Further research and awareness about such rare malignancies are crucial to improve the management of similar cases in the future.
